# Community Knowledge, Attitude, and Practices Related to Strabismus and Strabismus Treatment and Surgery in Al-Jouf Region, Saudi Arabia

**DOI:** 10.7759/cureus.50960

**Published:** 2023-12-22

**Authors:** Bader Alanazi, Abdulmohsen Almulhim, Abdulrahman Alfaleh, Rana Amsaiab, Alhanof Ahmed Althari, Rasha Alashjaee, Rahaf Hamdan Alsabilah, Ohoud Mohammed F Alsahli

**Affiliations:** 1 Ophthalmology Department, College of Medicine, Jouf University, Sakakah, SAU; 2 Medicine, Jouf University, Sakakah, SAU

**Keywords:** strabismus treatment and surgery, strabismus risk factors, strabismus, practice, attitude, knowledge

## Abstract

Background: Strabismus is the misalignment of the visual axis of both eyes caused by abnormalities in binocular vision or anomalies of neuromuscular control of ocular motility. This study aimed to assess the community knowledge, attitude, and practices related to strabismus and strabismus treatment and surgery in the Al-Jouf region of Saudi Arabia.

Methods: This study utilized a quantitative cross-sectional design. The consented volunteering adult participants were randomly selected through sequential enrollment to the completion of the target sample size from five population groups constituting the Al-Jouf region of Saudi Arabia. The data were collected through an online validated self-administered questionnaire. The collected data were analyzed using descriptive and inferential statistics.

Results: The study included 340 participants; 67.6% of them were females and 32.4% were males. Only 66.8% of participants correctly defined strabismus. Symptoms of strabismus were identified as double vision (58.5%), blurred vision (48.2%), headache (31.8%), eye fatigue (41.5%), and difficulty reading (47.1%). Regarding risk factors of strabismus, 48.5% reported family history, 39.4% reported uncorrected refractive errors, and 23.2% reported long screen time. Complications of untreated strabismus were reported as low self-confidence (39.1%), vision loss (27.6%), poor interpersonal relationships (25.9%), amblyopia (42.6%), and cosmetic stigma (14.1%). Only 45.6% of the participants agreed to marry someone with strabismus or allow their relatives to do so. The majority of participants (72.6%) would advise a strabismus patient to visit an ophthalmologist. Despite a higher knowledge regarding strabismus among females, younger agers, higher education, single persons, workers/students, and those inhabiting the capital city of the region, such association did not reach significance.

Conclusion: The study shows a moderate level of knowledge, attitude, and practices among the general population of Al-Jouf Saudis toward strabismus. There was no significant relationship between knowledge of strabismus and any of the demographic characteristics of the participants. This might prove detrimental to the early detection and treatment of strabismus to prevent its complications and improve its outcomes and the quality of patients' lives. Institution of correlation plans are mandated by the Ministry of Health and interested stakeholders.

## Introduction

Strabismus is a common childhood disorder that causes deviation or squinting of eyes. If left untreated, it can permanently affect the vision, personality, and mindset of the child [1]. The overall prevalence of strabismus, exotropia, and esotropia is 1.93%, 1.23%, and 0.77%, respectively, altogether reaching 6% globally. The prevalence of strabismus is 5% in developed countries among children less than five years [2]. The deviation can be either steady or intermittent, twisted in (incyclotropia), rotated out (excyclotropia), turned in (esotropia, ET), turned out (exotropia, XT), turned down (hypotropia), turned up (hypertropia), and so on, depending on the fusional position. The most prevalent type of deviation in the recorded clinical studies worldwide is the horizontal deviation, i.e., XT and ET [1].

Refractive errors, a family history of strabismus, and a history of maternal smoking during pregnancy are all risk factors for strabismus. It can have a negative impact not only on the binocular single vision, such as simultaneous perception, fusion, and stereopsis, but also on cosmetic presentation, which can have serious psychological effects [3]. As regards complications, strabismus is a common cause of amblyopia in youngsters, which can last into adulthood if not addressed promptly. As a result, strabismus and amblyopia, particularly in preschool children, must be identified and treated early to maximize binocular potential and enhance outcomes [4,5]. The basic goal of strabismus treatment is to re-establish appropriate ocular alignment, treat amblyopia, maintain binocularity, and eliminate diplopia. Treatment includes observation, refractive error correction, orthoptics, prismatic correction, pharmaceutical therapy, i.e., "miotics," botulinum toxin, and extraocular muscle surgery [6]. The aesthetic acceptability of surgery for horizontal and bilateral strabismus has improved and the overall surgical success rate for horizontal strabismus surgery is very high. However, with partially accommodative esotropia, surgical success was favorably connected with younger age at presentation and the lack of amblyopia [3].

The level of knowledge and awareness of parents is strongly related to the stress, both psychological and social, experienced by children with strabismus and their parents, where the more educated parents are more observant [7]. Parents may put off treatment since they are ignorant of the vision and stereoscopic impairment that strabismus can cause. Community-level health education regarding strabismus and its impacts is critical for preventing strabismic amblyopia and its associated psychological effects [8]. A previous study, conducted in our target area, showed that strabismus was properly identified by 52.8% of the 589 participants, and 71.5% of them said that strabismus is treatable. Age, gender, employment status, and educational achievement were also found to be statistically significantly connected with the awareness of strabismus treatability. The majority of participants were aware of the risk factors and consequences of strabismus [9]. Another Saudi study revealed that half of the participants had a good awareness of many strabismus-related topics. However, the study also highlighted some detrimental misconceptions about the psychosocial and economic repercussions of strabismus. It is interesting to note that while there is a high degree of awareness about the many strabismus traits, there are still gaps in knowledge regarding its psychosocial and economic repercussions [3]. It is critical to assess the socio-demographic distribution, environmental risk factors, and community understanding to control strabismus [5]. Gaps of knowledge and discrepancies among the reported studies could be attributed to differences in population traditional, genetic, and demographic background, and the assessment tools [3].

The discrepancies among the reported studies highlight the importance of conducting more studies to assess the community's knowledge, attitude, and practices related to strabismus and its treatment. This is particularly important in regions such as Al-Jouf, Saudi Arabia, where there may be limited access to information and resources. This study aimed to evaluate the awareness and behavior of the community in the Al-Jouf region of Saudi Arabia regarding strabismus and its treatment. Accordingly, healthcare providers can better tailor their education and outreach efforts to address any misconceptions or barriers to seeking treatment for better outcomes.

## Materials and methods

Setting and participants

The present online questionnaire-based cross-sectional study was conducted in the Al-Jouf region of Saudi Arabia. The questionnaire was opened to all community sectors to consider all volunteering consented adult residents within the region, including employees, householders, university students, workers, and educated individuals. Data collection was done during the academic year 2023 and the study spanned six months for the five population groups constituting the region, i.e., Sakaka (the capital city), Dumat-Al-Jandal, Tabarjal, Al-Qurayyat, and the scattered smaller populations. The questionnaire was distributed through social media platforms after securing ethical approval from the Local Research Ethics Committee, Jouf University, Sakaka, Saudi Arabia. The sample size was determined to be 300 using the Survey System automated calculator (https://www.surveysystem.com/sscalc.htm), at a confidence level of 95% and a margin of error of 5%. We collected data from 340 participants to guard against invalid and incomplete surveys. Randomization was ensured through the sequential consideration of the arrival of the complete survey to the target sample size.

Data collection tool

An open-source and previously validated questionnaire, in the national Arabic language, on knowledge, attitude, and practices regarding strabismus and strabismus treatment and surgery was utilized for data collection [10]. This questionnaire consisted of six sections, covering all aspects of data collection, including brief summary of the study, the investigating team, and the participant's role, the participant’s consent, un-identifying participant’s sociodemographic data, and close-ended questions about awareness of eye diseases, perceptions of strabismus and its treatment, attitudes toward strabismus, and practices regarding people with strabismus.

Statistical analysis

The data analysis was conducted using SPSS Statistics for Windows version 21 (IBM Corp., Armonk, NY). The level of significance was set at P < 0.05.

## Results

Table 1 presents the sociodemographic characteristics of participants. The study included 340 participants; 67.6% of them were females and 32.4% were males. The age distribution shows that the majority (59.4%) of the population falls within the 18-30 years age range. A major portion of the population was single (53.2%), married individuals made up 42.1%, while divorced and widowed individuals constituted 3.2% and 1.5% of the study population, respectively. In terms of educational status, the majority (79.4%) of the population has attained a university undergraduate or postgraduate degree. Individuals with a secondary school education constituted 19.4%, while 0.6% of the population were uneducated. Workers and students were the two largest occupational groups, representing 39.4% and 41.8% of the population, respectively. Unemployed individuals and retirees made up smaller percentages of 15.6% and 3.2%, respectively. The regional distribution indicates that Sakaka was the most populous region (51.2% of the population), followed by Dumat Al-Jandal at 32.4%, and Al-Qurayyat at 12.6%. Other regions made up a smaller percentage of 3.8%.

**Table 1:** Sociodemographic characteristics of participants assessed for their knowledge, attitude, and practices related to strabismus (n = 340). Data are presented as frequencies (n and %).

Characteristics		n	%
Gender	Male	110	32.4
	Female	230	67.6
Age, years	18-30	202	59.4
	31-40	41	12.1
	41-50	53	15.6
	>50	44	12.9
Marital status	Single	181	53.2
	Married	143	42.1
	Divorced	11	3.2
	Widow	5	1.5
Educational status	Uneducated	2	0.6
	Primary school level	2	0.6
	Secondary school level	66	19.4
	University undergraduate/postgraduate	270	79.4
Job	Worker	134	39.4
	Student	142	41.8
	Unemployed	53	15.6
	Retired	11	3.2
Region	Al-Qurayyat	43	12.6
	Dumat Al-Jandal	110	32.4
	Sakaka	174	51.2
	Tabarjal	2	0.6
	Other regions	11	3.2

**Figure 1:**
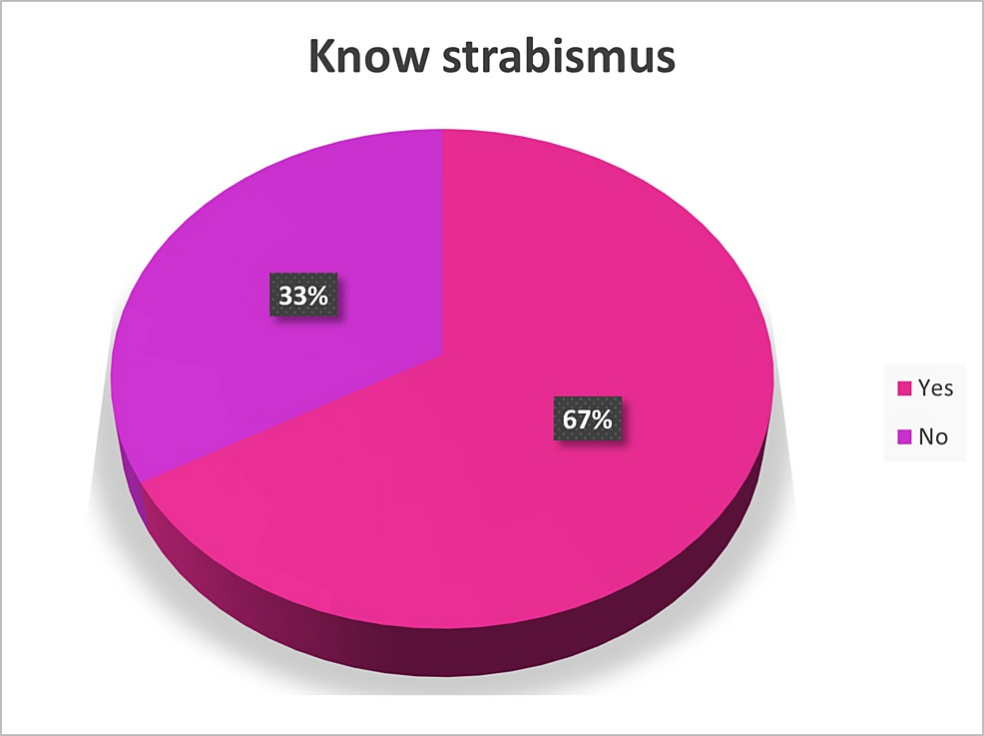
Participants’ knowledge of the definition of strabismus.

Only 66.8% of the participants correctly defined strabismus (Figure 1).

As presented in Table 2, the most common symptoms of strabismus were identified by participants as difficulty reading (47.1%), double vision (58.5%), blurred vision (48.2%), headache (31.8%), eyestrain (41.5%), and loss of depth perception (23.2%). Risk factors for strabismus were identified as family history (48.5%), uncorrected refractive errors (39.4%), head injuries (28.2%), very long screen time (23.2%), systemic disease (12.9%), tumor (12.1%), low social and economic status (4.1%), and having a mother who smokes (4.4%). The most common complications reported in the data were low self-confidence (39.1%), amblyopia (42.6%), vision loss (partial or complete) (27.6%), poor interpersonal relationships (25.9%), and cosmetic stigma (14.1%). The data show strabismus surgeries identified by participants as weakening procedures (16.2%), vector adjustment procedures (22.6%), and strengthening procedures (29.1%).

**Table 2:** Participants’ knowledge regarding strabismus (n = 340). Data are presented as frequencies (n and %).

Parameter		n	%
Symptoms of strabismus (bias risk)	Difficulty reading	160	47.1
	Double vision	199	58.5
	Blurred vision	164	48.2
	Headache	108	31.8
	Eyestrain	141	41.5
	Loss of depth perception	79	23.2
	I don't know	51	15.0
Risk factors that may lead to strabismus (bias risk)	Family history	165	48.5
	Uncorrected refractive errors	134	39.4
	Head injuries	96	28.2
	Squint	106	31.2
	Very long screen time	79	23.2
	Systemic disease	44	12.9
	Tumor (retinoblastoma)	41	12.1
	Low social and economic status	14	4.1
	Smoker mother during pregnancy	15	4.4
	I don't know	66	19.4
Strabismus surgery is	Weakening actions: Reduce the effective force of muscle action	55	16.2
	Vector modification procedures: Change the direction of muscle action (transformational)	77	22.6
	Strengthening actions: Enhance the pulling of the muscle	99	29.1
	Strabismus cannot be treated with surgery	18	5.3
	I don't know	154	45.3
Complications of untreated strabismus	Low self-confidence	133	39.1
	Vision loss (partial or complete)	94	27.6
	Poor interpersonal relationships	88	25.9
	Eye laziness	145	42.6
	Cosmetic stigma	48	14.1
	Strabismus does not cause any complications	33	9.7
	I don't know	79	23.2

**Figure 2:**
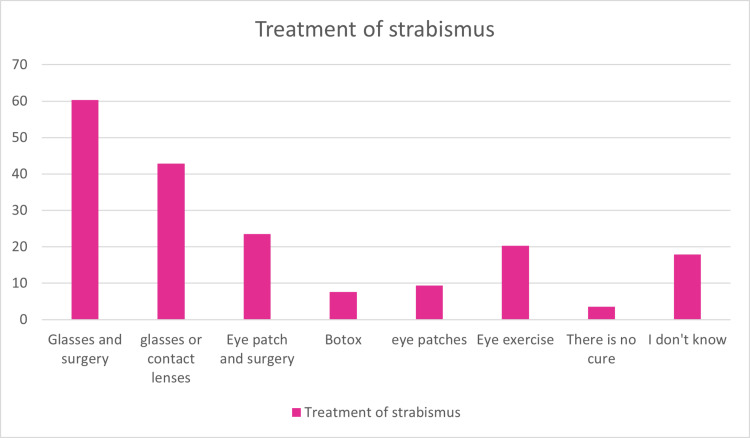
Participants’ knowledge of the treatment of strabismus. Data are presented as %.

As for treatment, 60.3% of the participants reported both eyeglasses and surgery, 42.9% reported glasses and contact lenses, 23.5% reported eye patch and surgery, and 20.3% reported eye exercises (Figure 2).

Regarding attitude, 52.4% reported being bullied because of having strabismus or for being a family member of a strabismus patient. While 45.6% of respondents indicated that they would be open to marrying someone with a squint, 54.4% expressed reluctance. Moreover, the data show that the majority of respondents (72.6%) would advise someone with squint to visit an ophthalmologist if they see them with the condition (Table 3).

**Table 3:** Participants’ attitude toward strabismus (n = 340). Data are presented as frequencies (n and %).

Item		n	%
As a patient with a squint or you have a relative with a squint, have you been bullied because of the squint?	Yes	178	52.4
	No	162	47.6
Would you marry someone with strabismus or allow your relatives to do so?	Yes	155	45.6
	No	185	54.4
What do you do if you see someone with a squint?	Advise him to visit an ophthalmologist	247	72.6
	Nothing can be done	93	27.3

Table 4 presents the association between the different demographic characteristics of participants and knowledge regarding strabismus. Higher knowledge was noted among females (46.2%) compared to males (20.6%), with no significant association. In terms of age, the data show that the highest knowledge of strabismus was among the 18-30 years age group (40.0%), followed by those >50 years of age (9.7%), with no significant association. Educational status also seems to play a role, as the knowledge of strabismus was higher among those with an undergraduate or postgraduate education (54.7%) compared to those with primary or secondary education levels. However, no significant association with educational level was noted. Occupation and region did not seem to have a significant impact on the knowledge of strabismus, as the percentages were relatively similar across different categories.

**Table 4:** Participants' knowledge of strabismus in association with their sociodemographic characteristics (n = 340).

		Know strabismus		Total (N = 340)	P-value
		Yes	No		
Gender	Male	70	40	110	0.397
		20.6%	11.8%	32.4%	
	Female	157	73	230	
		46.2%	21.5%	67.6%	
Age	18-30	136	66	202	0.388
		40.0%	19.4%	59.4%	
	31-40	27	14	41	
		7.9%	4.1%	12.1%	
	41-50	31	22	53	
		9.1%	6.5%	15.6%	
	>50	33	11	44	
		9.7%	3.2%	12.9%	
Marital status	Single	124	57	181	0.176
		36.5%	16.8%	53.2%	
	Married	96	47	143	
		28.2%	13.8%	42.1%	
	Divorced	4	7	11	
		1.2%	2.1%	3.2%	
	Widow	3	2	5	
		0.9%	0.6%	1.5%	
Educational status	Illiterate	1	1	2	0.230
		0.3%	0.3%	0.6%	
	Primary	2	0	2	
		0.6%	0.0%	0.6%	
	Secondary	38	28	66	
		11.2%	8.2%	19.4%	
	Undergraduate, post-graduate	186	84	270	
		54.7%	24.7%	79.4%	
Occupation	Worker	89	45	134	0.662
		26.2%	13.2%	39.4%	
	Student	99	43	142	
		29.1%	12.6%	41.8%	
	Unemployed	32	21	53	
		9.4%	6.2%	15.6%	
	Retired	7	4	11	
		2.1%	1.2%	3.2%	
Region	Al Qurayyat	26	17	43	0.741
		7.6%	5.0%	12.6%	
	Dumat Al-Jandal	74	36	110	
		21.8%	10.6%	32.4%	
	Sakaka	117	57	174	
		34.4%	16.8%	51.2%	
	Other	10	3	13	
		3%	0.9%	3.9%	

## Discussion

A better prognosis can result from early detection and treatment of strabismus, with a good perception as a prerequisite. As a result, parents' and the general public's ignorance has a negative impact on the outcomes of strabismus [11,12]. The present study aimed to assess the community knowledge, attitude, and practices related to strabismus, its treatment and surgery, and its risk factors in the Al-Jouf region of Saudi Arabia. We observed correct identification of strabismus among the majority of participants (66.8%). The finding was in agreement with a Saudi study [6] and was higher compared to another one presenting 50.6% [3]. Regarding risk factors of strabismus, 48.5% of the participants reported family history, 39.4% reported uncorrected refractive errors, and 23.2% reported too long screen time. According to a prior study, the majority of participants were aware of the strabismus risk factors, with family history and eye refractive abnormalities as the most frequently cited risk factors [13]. The most frequent risk factors for strabismus, according to other publications, were inherited factors and ocular illnesses [14,15].

Strabismus is subject to a variety of interventions and therapies. The main goal of treatment is to align the visual axis. Conservative solutions include prisms to realign the visual axes and orthoptic exercises to encourage and establish binocular control of ocular alignment, where both eyes can later act as a pair. Surgery to permanently change extraocular muscle activity and, consequently, ocular alignment. Botulinum toxin injection, to specific extraocular muscles, is an invasive therapy option [16]. Botulinum toxin temporarily paralyzes the extraocular muscle and alters ocular alignment. It normally takes two to three months for the altered ocular alignment to resolve [17]. This strategy is thought to be challenging to use in youngsters since it necessitates anesthesia and can cause difficulties in case the toxin leaks into the muscle that raises the eyelid, the levator palpebrae superioris, causing a droopy upper lid (ptosis) [18,19]. In our study, 60.3% of participants reported both eyeglasses and surgery as treatment of strabismus, 42.9% reported glasses and contact lenses, and 23.5% reported eyepatch and surgery, while 7.6% reported Botox injection.

Complications of untreated strabismus were reported by our participants to be low self-confidence (39.1%), vision loss (27.6%), poor interpersonal relationships (25.9%), amblyopia (42.6%), and cosmetic stigma (14.1%). In comparison, a previous study reported sight loss (4.6%), cosmetic stigma (3.9%), and poor self-image as consequences of uncorrected strabismus, while the majority of respondents selected "All of the above" [13]. An overwhelming majority (95%) of participants in another study reported psychological difficulties as the complication, regardless of the kind of strabismus [20]. Children with strabismus found it difficult to make friends and get employment, according to a cross-sectional study conducted in India [7]. On the other hand, children who underwent strabismus surgery had improved physically and psychologically [21]. Other vision-related issues associated with strabismus could be financially burdensome [15]. Ethiopian participants highlighted visual loss (43.1%), disfigurement (15.7%), psychological anguish (8.3%), and difficulty in getting married (4.3%) as complications of untreated strabismus [22].

Regarding attitude, only 45.6% of our participants would marry someone with strabismus or allow their relatives to do so. However, 72.6% of the participants would advise a strabismus patient to visit the ophthalmologist. In comparison, among Ethiopian participants, 51.4% of respondents said they would not want to marry someone who has strabismus or permit the marriage of a relative to one, as they consider them disabled (38.2%), look unattractive (22.1%), or afraid of having children who look like them (21.6%), while 17.2% did not want to state a reason [22].

Despite a higher knowledge regarding strabismus among females, younger agers, higher education, single persons, workers/students, and those inhabiting the capital city of the region, our results did not present significant correlation between the corrected identification of strabismus vs. any of the studies' sociodemographic characteristics of the participants. This was a surprising observation as other studies, even assessing the same population, reported significant association. For example, age, gender, employment status, and educational achievement had a significant connection with the awareness of strabismus treatability [9], and the Ethiopian study reported such a relationship vs. each of gender, age, education, or occupation status [13]. However, the Nigerian population expressed no association between age and knowledge of strabismus, with more knowledge among females [14]. As would be expected, another Ethiopian study found that awareness of strabismus was substantially correlated with employment and monthly income [8]. Three studies, an Ethiopian, a Saudi, and a Colombian, reported a positive significant association with education and older age [23-25].

The poor knowledge and attitude performance of our target population was surprising to us, particularly in light of previously reported better knowledge, attitude, and practice scores from the same region. Online survey distribution through social media platforms is liable to bias in the balanced representation of all sectors of the community depending on personal interests in and accessibility of such media. In addition, the study was limited to Al-Jouf, Saudi Arabia, which hinders generalizing our findings to a large population of Saudi Arabia. However, since other studies also utilized online surveys, it is advisable that bigger studies that utilize stratification of the population with face-to-face interview type of participation and data collection are mandated.

## Conclusions

The findings of the study indicate a moderate level of knowledge and attitude toward strabismus among the general population of Al-Jouf, Saudi Arabia. It is concerning that there was no significant relationship between knowledge of strabismus and sociodemographic factors of the participants, despite a higher knowledge regarding strabismus among females, younger agers, higher education, single persons, workers/students, and those inhabiting the capital city of the region. This could have negative implications for the eye and vision health, psychosocial well-being, and quality of life of individuals with strabismus, as well as its complications and outcomes. It is imperative to address misconceptions and minimize the adverse effects of strabismus through targeted health education followed by longitudinal studies to assess the effectiveness of interventions aimed at raising awareness and reducing the stigma associated with strabismus.

## References

[1] Tegegne MM, Fekadu SA, Assem AS (2021). Prevalence of strabismus and its associated factors among school-age children living in Bahir Dar city: a community-based cross-sectional study. Clin Optom (Auckl).

[2] Hashemi H, Pakzad R, Heydarian S (2019). Global and regional prevalence of strabismus: a comprehensive systematic review and meta-analysis. Strabismus.

[3] Alzuhairy S, Alabdulrazaq ES, Alharbi IM, Alharkan DH (2019). Knowledge and attitude towards strabismus among parents of Saudi children with strabismus. Int Surg J.

[4] He H, Fu J, Meng Z, Chen W, Li L, Zhao X (2020). Prevalence and associated risk factors for childhood strabismus in Lhasa, Tibet, China: a cross-sectional, school-based study. BMC Ophthalmol.

[5] Dakroub M, El Hadi D, El Moussawi Z, Ibrahim P, Al-Haddad C (2022). Characteristics and long-term surgical outcomes of horizontal strabismus. Int Ophthalmol.

[6] Khojah MS, Al-Ghamdi S, Alaydarous S, Homsi JJ, Alhasan A, Alsubaie S, Alhibshi N (2020). Knowledge and attitude toward strabismus in Western Province, Saudi Arabia. Cureus.

[7] Singh A, Rana V, Patyal S, Kumar S, Mishra SK, Sharma VK (2017). To assess knowledge and attitude of parents toward children suffering from strabismus in Indian subcontinent. Indian J Ophthalmol.

[8] Assaye AK, Tegegn MT, Assefa NL, Yibekal BT (2023). Knowledge towards strabismus and associated factors among adults in Gondar town, Northwest Ethiopia. J Ophthalmol.

[9] Alnuman RWS, Alhablani FSN, Alruwaili EMA, Alruwaili GAA, Alruwaili RR, Alruwaili RMJ, Zaky KAE (2021). Prevalence of squint in primary school children and its associated sociodemographic factors in Sakaka City, Aljouf Region, Saudi Arabia. IJMDC.

[10] Alzahrani N, Alhibshi N, Bukhari D, Alijohani M, Madani F (2018). Awareness, perceptions and knowledge of amblyopia among pediatrics and ophthalmology clinics attendees in King Abdulaziz University Hospital, Jeddah. Int J Adv Res.

[11] Kanukollu VM, Sood G (2023). Strabismus. StatPearls [Internet]. Treasure Island (FL): StatPearlsPublishing.

[12] Johns HA, Manny RE, Fern KD, Hu YS (2005). The effect of strabismus on a young child's selection of a playmate. Ophthalmic Physiol Opt.

[13] Alshammari TM, Alamri KK, Ghawa YA, Alohali NF, Abualkol SA, Aljadhey HS (2015). Knowledge and attitude of health-care professionals in hospitals towards pharmacovigilance in Saudi Arabia. Int J Clin Pharm.

[14] Isawumi MA, Ulaikere M, Adejumo OO, Adebayo M, Kekunnaya R (2014). Awareness, perceptions and knowledge of strabismus among patients visiting a tertiary eye clinic in Southwest Nigeria. Int Ophthalmol.

[15] Bukhari D, Alhibshi N, Alzahrani N, Aljohani M, Madani F (2018). Awareness, perceptions and knowledge of strabismus among pediatrics and ophthalmology clinics attendees in King Abdulaziz University Hospital, Jeddah. Ann Int Med Den Res.

[16] Rowe FJ, Noonan CP (2017). Botulinum toxin for the treatment of strabismus. Cochrane Database Syst Rev.

[17] Scott AB (1980). Botulinum toxin injection into extraocular muscles as an alternative to strabismus surgery. J Pediatr Ophthalmol Strabismus.

[18] Rowe FJ, Noonan CP (2012). Botulinum toxin for the treatment of strabismus. Cochrane Database Syst Rev.

[19] Rowe FJ, Noonan CP, Nayak H (2005). Botulinum toxin as a treatment option for decompensating intermittent strabismus in children. Transactions of the 30th European Strabismological Association.

[20] Enzenauer RW (1994). Strabismus repair is not “cosmetic”. J Pediatr Ophthalmol Strabismus.

[21] Ziaei H, Katibeh M, Mohammadi S (2016). The impact of congenital strabismus surgery on quality of life in children. J Ophthalmic Vis Res.

[22] Geta K, Bejiga A (2011). Knowledge, attitude and practice towards strabismus in Cheha District, Central Ethiopia. Ethiop J Health Dev.

[23] Alemayehu HB, Tsegaye KB, Ali FS, Adimassu NF, Mersha GA (2022). Knowledge and attitude towards strabismus among adult residents in Woreta town, North West Ethiopia: a community-based study. PLoS One.

[24] Mabrouk AMK, Alshammari HM, Alshammari RF (2021). Awareness, perceptions and knowledge of strabismus among Ha’il population, KSA. World Fam Med J.

[25] Diaz-Quijano FA, Martínez-Vega RA, Rodriguez-Morales AJ, Rojas-Calero RA, Luna-González ML, Díaz-Quijano RG (2018). Association between the level of education and knowledge, attitudes and practices regarding dengue in the Caribbean region of Colombia. BMC Public Health.

